# The meaning of ‘acceptance’ of a psychiatric diagnosis: qualitative study of illness narratives with review of the literature

**DOI:** 10.1192/bjo.2025.10810

**Published:** 2025-09-04

**Authors:** Magali J. de Rooy, Megan M. Milota, Stefan M. van Geelen, Léon C. de Bruin, Floortje E. Scheepers

**Affiliations:** Department of Psychiatry, University Medical Centre Utrecht, The Netherlands; Department of Bioethics and Health Humanities, Julius Centre for Health Sciences and Primary Care, University Medical Centre Utrecht, The Netherlands; Education Centre, University Medical Centre Utrecht, The Netherlands; Department of Anatomy & Neurosciences, Amsterdam University Medical Centre, The Netherlands

**Keywords:** Psychiatric diagnosis, psychiatric classification, acceptance, narratives

## Abstract

**Background:**

Although diagnosis acceptance is frequently discussed in psychiatric practice and research, both components – psychiatric diagnoses and the act of accepting them – are inherently unclear.

**Aims:**

The present study aimed to evaluate how well existing theoretical concepts of diagnosis acceptance align with patient experiences and to explore themes related to diagnosis acceptance.

**Method:**

An iterative thematic analysis was conducted on 30 illness narratives from (former) psychiatric patients. The analysis proceeded through three phases: (a) review of transcripts for overall narratives and attitudes toward diagnoses, (b) extraction of detailed data using a narrative summary template and (c) refining and comparison of themes across narratives.

**Results:**

Existing acceptance theories insufficiently captured the lived experiences reflected in the narratives. Attitudes toward diagnoses were multidimensional, fluctuated over time and were often described using terms other than ‘acceptance‘. Participants emphasised the importance of sharing their stories without being defined by a diagnosis and conflated DSM-5 classifications with broader diagnostic terms, highlighting challenges in communication of psychiatric constructs. Disagreement with diagnoses did not necessarily hinder therapeutic relationships, emphasising the importance of collaboration over consensus.

**Conclusions:**

Given the limited practical application of existing acceptance theories and our findings on contextual factors relevant to psychiatric diagnosis attitudes, the necessity of diagnosis acceptance as a stand-alone goal for positive outcomes should be questioned. Rather than imposing classifications, creating co-constructed narratives may be more effective. Researchers and clinicians are encouraged to adopt narrative approaches to better understand and support patients, thereby fostering reciprocal, patient-centred mental healthcare.

‘The verbal act of presenting a patient with a diagnosis is never a simple act of conveying value-neutral biomedical information. It is an act fraught with symbolism’, said linguist and terminal diagnosis recipient Suzanne Fleischmann, and this seems especially true for the field of psychiatry.^1^ Patients’ ‘acceptance’ or ‘non-acceptance’ of a diagnostic classification is a prominent topic in both research^2–5^ and clinical practice recommendations.^3,6–8^ Research suggests that a significant portion of the therapeutic effect of psychiatric treatment stems from the patient–clinician relationship, and that discordance in this relationship can have significant repercussions, underscoring the importance of consensus in both diagnosis and treatment plan.^9–11^ While at first glance the meaning of acceptance of a psychiatric diagnosis might seem obvious, perceptions, beliefs and opinions vary widely among patients, clinicians and researchers, and many different interpretations of both ‘psychiatric diagnosis’ and acceptance exist.^12–14^ Notably, regardless of the high frequency with which the term is used, it is not commonly understood what acceptance in this context actually means, raising doubts about the utility of the concept.

Many authors discuss the complex and multifaceted concept of diagnosis acceptance yet fail to acknowledge that both components – the personal meaning of psychiatric diagnoses and the act of accepting them – are inherently unclear. Additionally, diagnosis attitudes are often discussed and studied without including patients’ perspectives. It is becoming increasingly clear that the current way of discussing acceptance of psychiatric diagnoses is neither well defined, evidence-based, practical nor reflective of the patient’s perspective. Diagnosis acceptance – arguably a subjective experience – should be studied in an inclusive way that takes individuals’ lived experiences into account. The present article therefore focuses on the question of how people that have received a psychiatric classification understand and discuss (topics adjacent to) diagnosis (non-)acceptance. Do prominent theoretical concepts from the literature on diagnosis (non-)acceptance capture people’s experiences and opinions? And what can we learn about attitudes towards diagnoses from illness narratives? In particular, the aim of our study was to investigate the main patterns and overarching themes concerning the various dimensions of psychiatric diagnosis acceptance, in order to establish whether the traditional definitions of acceptance sufficiently reflect patients’ perspectives and experiences. A deeper understanding of these themes fosters greater mutual understanding, which generally leads to more fruitful collaboration between clinicians and patients.

Our study consisted of a qualitative analysis of illness narratives by (former) psychiatric patients, shared within the setting of the Dutch ‘Psychiatry Story Bank’.^15^ First, we used the main theoretical concepts related to diagnosis acceptance and tested whether they are recognisable, appropriate and applicable to the narratives – a process referred to as ‘sensitising concepts’. Second, we studied the narratives for emerging themes related to people’s attitude towards their diagnosis and (non-)acceptance. The traditional concepts of (non-)acceptance turned out to be insufficient at capturing the meaning within the narratives. Particular attention is required for the various dimensions of diagnosis towards which attitudes may be directed – such as the experienced symptoms versus societal consequences of a diagnosis – as well as individual preferences in contextualising and narrating the diagnosis. Based on the results, we have formulated recommendations for both future research and clinical practice.

## Method

### Setting and participants

This study used data previously collected from the Psychiatry Story Bank from the University Medical Centre Utrecht (The Netherlands) in the form of anonymised interview transcripts.^15^ In this project, illness narratives of (former) psychiatric patients, their relatives or partners and professionals are collected by means of semi-structured interviews, and analysed with the aims of gaining insight into lived experience and improving personal recovery. A second aim of the Psychiatry Story Bank is to counter epistemic injustice by ensuring that participants have full control over how they share their experiences. The authors assert that all procedures contributing to this work comply with the ethical standards of the relevant national and institutional committees on human experimentation, and with the Helsinki Declaration of 1975 as revised in 2013. All procedures involving human subjects/patients within the Psychiatry Story Bank project were evaluated by The Medical Ethical Review Committee of the University Medical Centre of Utrecht, who confirmed that the Dutch Medical Research Involving Human Subject Act (WMO) did not apply. Subsequently, official approval of this study by the Medical Ethical Review Committee was not required (reference no. WAG/mb/16/030724). Written informed consent was obtained from all participants. All participants were diagnosed in the Netherlands while DSM-III, DSM-IV or DSM-5 were in use. Further details about the project can be found in papers by van Sambeek and colleagues.^16,17^ From all 173 interviews with (former) patients (collected between January 2017 and January 2024), 30 (17.3%) transcripts were randomly selected and numbered 1 to 30. In the case of interviews that are displayed as vignettes, interviewees were assigned a pseudonym.

### Research design

A phenomenological and interpretative approach informed the study design and analysis. We performed an iterative thematic analysis of stories from people with one or multiple psychiatric classifications. We began by compiling the most prominent concepts or theories related to psychiatric diagnosis (non-)acceptance, which we found by studying existing literature on acceptance and diagnosis acceptance (see Theoretical concepts, below). The literature was not reviewed in a systematic manner, meaning that this overview is therefore not exhaustive. We chose to draw on acceptance models from chronic illness and pain research, because our aim was to engage with broadly established theoretical frameworks rather than to validate findings from narrowly focused studies in specific psychiatric populations that have adopted particular assumptions about diagnosis acceptance into their research design. The theoretical concepts served as a starting point for the interpretation of our qualitative data, specifically remarks concerning people’s acceptance of their diagnosis.^18,19^ We aimed to assess – or sensitise – the utility of these concepts in capturing people’s (non-)acceptance of their psychiatric diagnoses. Next, we investigated how individuals speak about diagnosis acceptance or related topics, and what explicit or implicit meaning is attached. Our approach involved pinpointing significant experiences related to diagnosis attitudes or acceptance within an interviewee’s story, comparing these across different parts of their narrative and then relating them to similar experiences shared by other interviewees. This comparative process helped uncover common themes, using the analysis of individual stories as a window into broader shared experiences related to the (non-)acceptance of psychiatric diagnoses.

### Data processing and analysis

Members of the research team (M.J.d.R. and M.M.M.) conducted close reading sessions of five randomly selected transcripts to compare and broaden their understanding of narratives and the ways people spoke about diagnosis acceptance. Based on this, M.J.d.R. created a ‘narrative summary template’ consisting of explorative questions, questions regarding the theoretical concept and summarising questions, thus building on previous templates.^20,21^ An English translation of the narrative summary template is available in Supplementary Materials available at https://doi.org/10.1192/bjo.2025.10810. The completed narrative summary templates contain a summary of the interview and specific quotes on attitudes towards diagnosis and acceptance, and related topics.

Our analysis of each of our 30 randomly selected transcript involved three phases. First, an initial reading provided a preliminary understanding of the overall narrative and the individual’s attitude towards their diagnosis. Second, data were extracted using the narrative summary template by the first author for all 30 transcripts, using double extraction in 20% of cases to validate the extraction template, following which sufficient intercoder reliability was established. Extracted fragments or interpretations about diagnosis attitudes were compared with the whole story. The narrative summary template was iteratively refined across multiple meetings between the authors, based on salient patterns and important findings, and previously analysed transcripts were updated as needed. Third, further analysis and interpretation of the data in the narrative summaries were conducted. Data in which the theoretical concepts were recognisable were compared with the larger narrative to determine applicability. Overarching themes were identified by comparing interviewees’ extracted data across the narrative summaries. Interpretations were validated against earlier findings, as well as the broader narrative. Weekly meetings and monthly team reviews ensured ongoing discussion and calibration; this iterative process created a circular validation loop, ensuring that the insights aligned across all stages.

### Reflexivity

Within the existing database, as much as possible we chose random sampling as a method to prevent both sampling and selection bias. Interpretations of the data were discussed regularly among the research team. To enhance transparency and rigour, researchers’ assumptions, reflections and disagreements were recorded in a logbook, providing insight into adaptations in the research process and researchers’ motivations. Due to the anonymised nature of the transcripts, a member check of the data was not performed. It is important to acknowledge that, given the broader aims of the Psychiatry Story Bank project, the interviews were not explicitly designed to explore diagnosis acceptance. This probably contributed to heterogeneity in narrative structure and content, thereby enhancing the overall narrative richness.

### Theoretical concepts of (non-)acceptance

This section will detail some of the theories on acceptance that are prominent in the current literature, of which the applicability will be sensitised in the Results, below. Described concepts are: acceptance and non-acceptance as a subset of principles according to Williams and Lynn;^7^ active and resigning acceptance;^3,22–27^ and acceptance as the final stage in a grieving process.^7,27–30^ The theoretical concepts are elaborated on in Table 1.

**Table 1:** Summary of theoretical concepts of (non-)acceptance

Concept	Brief summary
Acceptance^7^	Non-attachment: not running towards, no attempt to control Non-avoidance: not running away from, remain present even for uncomfortable experiences Non-judgement: abstention from categorising experience as good or bad, right or wrong Tolerance: voluntarily tolerate experiences as they come while remaining present and without resignation Willingness: comply out of free choice, adaptive behaviour
Non-acceptance^7^	Denial: ignoring the reality of a situation in order to avoid anxiety Avoidance: unwillingness to remain in contact with situations, activities, environments, individuals, experiences or feelings that are uncomfortable Escape: fleeing from a situation, thing or experience Prejudice: holding negative convictions towards situations, things or experiences Non-compliance: unwillingness to comply with a proposed course of action
Active acceptance^3,22–27^	Characterised by incorporating the disease into daily life, without allowing it to dominate affairs completely
Resigning acceptance^3,22–27^	Characterised by giving up many activities and roles, and allowing the disease to take central control of life
Acceptance as a process of grief^7,27–30^	Receiving a diagnosis is often equated with grief, and acceptance then described as the final stage in a stage model of dealing with negative or shocking events

## Results

### Primary readings

The primary readings resulted in a general understanding of the overall structure and content of the narratives. While some stories were structured chronologically as a traditional story, others focused on specific experiences of the narrator, omitting other aspects or events. Generally, people spoke about their experiences of having a psychiatric diagnosis and their fluctuating attitude towards it in at least part of the story;. Twenty-one of the 30 narratives provided substantial and concrete material on diagnosis acceptance; of the nine that did not, five discussed (not) accepting specific experiences related to their diagnosis. While several people had a predominantly negative attitude towards their diagnosis or explicitly disagreed with it, almost all interviewees agreed with their healthcare professional (HCP) about having some sort of problem and a need for help, treatment or guidance. Descriptive characteristics of the interviewees can be found in Supplementary Materials.

### Concept analysis

The first part of the analysis consisted of analysing the texts according to the theoretical concepts (Table 1). We found elements of Williams and Lynn’s concepts (acceptance, non-acceptance or both) in 29 out of 30 transcripts. The other concepts of active and resigning acceptance and acceptance as a process of grief were to some extent recognisable in the transcripts. However, it was often unclear whether these concepts applied to someone’s attitude towards their diagnosis, or whether these facets were related to life events in general (e.g. feeling grief and entering a grieving process due to tragic/traumatic life events, not the diagnosis specifically). Segments from the narratives required so much interpretation by the researchers to fit these concepts that we deemed them unapplicable to the interview data.

Table 2 shows two narrative vignettes based on two interviews from our data-set. Characteristics of the persons, a summary of their narrative and analyses according to the chosen theoretical concepts (specifically aimed at capturing acceptance of the diagnosis, or DSM-5 classification itself, rather than other experiences, and further discussed below in Classification versus diagnosis) are shown. In interviewee Eric’s story, he gradually learnt to accept his mental problems once his healthcare provider had given it a name. In this story, the theoretical concepts help in explaining Eric’s attitude and capturing the sentiment of his narrative. For interviewee Laura, someone who changed from being diagnosis compliant to seriously questioning it and even requesting that it be removed from her patient file, this process was more complex. Trying to capture Laura’s attitude in non-acceptance concepts, her current attitude might be labelled as ‘denial’, ‘avoidance’ or ‘escape’. However, taking the context of her whole story into account, this does not leave room for her opinion or interpretation of the situation. Assuming that Laura’s view of her symptoms is accurate, she currently does not fit the criteria of the classification of a bipolar disorder, making her denial understandable. Furthermore, it became apparent that even someone like Laura, who is clearly non-accepting, could still comply with some of the acceptance concepts while simultaneously not complying with all the non-acceptance concepts. The opposite was also found to be true. In another case, the interviewee said that his autism had become a ‘special interest’ for him since his diagnosis. Nonetheless, reading his whole narrative, it cannot be said that he was non-attached to his diagnosis, i.e. he did not comply with all topics of acceptance yet his attitude seemed overwhelmingly accepting.

**Table 2:** Narrative vignettes of two interviews

	Eric (transcript no. 10)	Laura (transcript no. 4)
Gender	Male	Female
Age	Middle-aged	Middle-aged
DSM-5 class.	Schizoaffective disorder	Bipolar disorder
Time since diagnosis	>5 years (exact time unclear)	18 years
Vignette	This person developed psychotic symptoms, came into psychiatric care and was told that he had experienced psychosis. During his next hospitalisation, many examinations were carried out: ‘But there wasn’t a whole lot to tell because I was a little bit schizophrenic, a little bit manic depressive and had a little bit of social problems.’ During a later hospitalisation, it became clearer: ‘… they said schizophrenia, and officially schizoaffective disorder, because they found out that I always became psychotic first before becoming manic’. It took a while to adjust to this knowledge, but he was able to accept this part of himself: ‘Yes. It’s just part of the deal for me. Illness. After three, four years, I started to accept it. Because you know what you have. Before that you can’t really have closure, because you don’t have a clear diagnosis. That’s a big difference. You can get into contact with peers, and are able to join a patient organisation.’ He speaks of his diagnosis as a functional tool that led towards more understanding, peer support and acceptance. Reflecting on his diagnosis and his turbulent life, he says: ‘Yes, it changed my position in society. If I would have finished my school I would have a good job, a house, things like that. So this is very different. But it has enriched my life. I do think that it enriched my life.’	This person came into contact with psychiatric care because of depressive symptoms. After developing manic symptoms while taking antidepressants, she was diagnosed with bipolar disorder. The diagnosis offered relief: ‘I thought: oh, I’m not being dramatic, there is actually something wrong with me’, as well as a clear prognosis: ‘It was clear to me, I was told: I had bipolar disorder. It would bother me my whole life, and I would need medication indefinitely’. The diagnosis seemed to be the reason that no one looked for further causes for her depressive symptoms (such as the traumatic events in her childhood), or potential treatments besides medication. Soon, this person started behaving like someone with a debilitating disorder (e.g. believing that she couldn’t do many things), read books about bipolar disorder, joined support groups about it and made it her life: ‘Every day, I was occupied with my mood. I started to live like … it was my, it became my life. That diagnosis became my life.’ After her own HCP felt at a loss with regard to her ongoing symptoms, someone in another hospital suggested discontinuing her medication and re-evaluating her in a state without medication. She realised that the idea of being sick and needing medication her whole life did not have to be true: ‘I felt … the more I reduced the medication, I felt very vulnerable … And I actually started feeling better and better despite that vulnerability. I thought well yes, but this is who I am.’ This marked the start of her recovery, and made her reconsider her past: ‘It made me think: what has actually happened to me all these years? That diagnosis kept me very, very small.’ She switched from being diagnosis and treatment compliant to someone who questioned the validity and veracity of the diagnosis, and more broadly the mental healthcare system: ‘To what extent is it a good idea, DSM, putting people in boxes, with the huge stigma that’s attached to it?’ She has been doing well for a number of years now, and enquired whether her diagnosis could be removed from her patient file. Her HCP thus far refused, wanting to be cautious due to having known her for only a short period: ‘I’m finding myself, reinventing myself. I don’t have symptoms anymore and I don’t use medication. Why would I still have bipolar disorder?’
Acceptance^12^	All of the concepts are recognisable, without any one being particularly prominent. He speaks about his diagnosis without clear emotion or intensity.	Past: non-attachment. As the diagnosis became her life, it seems incorrect to say that there was non-attachment. Other concepts: fitting Present: non-attachment. She is not attached to the diagnosis, but that does not mean she accepts it. Tolerance: while she expresses wanting to have the diagnosis removed, she tolerates that this isn’t the case right now. Other concepts: not applicable
Non-acceptance^12^	Not applicable	Past: prejudice. Negative view of herself as someone very ill, with very limited capabilities. Other concepts: not applicable Present: unclear whether wanting to have the diagnosis removed should be classified as ‘denial’, ‘escape’, ‘avoidance’ or none of these. Non-compliance is applicable: does not accept the diagnosis as ‘true’ and regularly asks whether it can be removed. Prejudice/negative conviction towards ‘diagnoses’ or ‘DSM’ as stigmatising.

HCP, healthcare professional.

Various elements of these theoretical concepts by Williams and Lynn were recognisable in all of the narratives. However, for almost all narratives these concepts were unable to fully and accurately capture the meaning of the narrative and reflect that person’s lived experience.

### Further analysis: emerging themes

Iterative readings and discussion of the data helped us identify a number of themes regarding dimensions of diagnosis acceptance that are not covered in traditional acceptance theory.

#### The object of acceptance

Dimensions and quotes related to the object of acceptance – the psychiatric diagnosis – are displayed in Table 3 and elaborated on below. A visual representation of the (not mutually exclusive) dimensions from which diagnosis attitudes may originate can be viewed in Supplementary Materials.

**Table 3:** Interviewees’ quotes that correspond to attitudes originating in different dimensions of diagnosis

Theme	Excerpt
Relevance of DSM-5 classification (important versus unimportant)	^a^ ‘That was an important year for me, because that’s when, with the autism diagnosis, I really solved the puzzle. A lot of small pieces falling into place. It gave me a feeling of calmness, security. If you would ask me, *name*, are you happy? Are you comfortable? Yes, it’s never been better than these past years. No longer the feeling of chasing something.’ (transcript no. 15)
	^b^ ‘In the end, of course it was post-traumatic stress.’ (transcript no. 20)
	^c^ Mentions only having ‘a susceptibility to psychosis’ and ‘my psychotic condition’ (transcript no. 7)
Level of attitude (individual diagnosis versus diagnostic (DSM) system)	^d^ ‘I got a label put on my head that I am narcissistic, and have borderline. My psychologist said “you shouldn’t look at it as such, you only have those traits”. I said: So? OCPD [obsessive compulsive personality disorder] has traits as well, and I recognise those more than that bullshit you wrote down. I don’t recognise myself at all. I’m not selfish.. (transcript no. 1)
	^e^ ’I think there’s nothing wrong with diagnoses; I think there’s something wrong with the standard policy that goes along with them because you have to look at what helps each individual. And if you really insist on labels, then please just use the right label [laughs].’ (transcript no. 28)
	^f^ ‘And then I got the diagnosis borderline. Well, it was written on the paper, while I myself think I have complex post-traumatic stress disorder. (…) I think the whole paradigm is completely outdated. They are just putting labels on anyone, really. Already waving their pills around.’ (transcript no. 30)
	^g^ ‘Well, I’m trying to move away from that diagnosis [bipolar disorder], that word now. And it is bipolar, for sure. Because there are the two, the ups and the downs.’ (transcript no. 2)
Dimension of experience of own diagnosis (internally versus externally originating experiences)	^h^ ‘I came out of my depression with that explanation for why I kept hitting walls, why I felt different from other people. Why it felt like I constantly had to reinvent the wheel. Why I thought, okay, for me, things don’t go the way they go for others. In the end, that was Asperger’s for me. Okay, your brain works a little different, your feelings – maybe not your brain, but your feelings work different from other people. So it’s not strange things are a little different for you.’ (transcript no. 13)
	^i^ ‘And one eating disorder is different from the next. They often have a specific protocol in a department, that they use with patient one, two, three, four, five, six, seven and eight, while patient eight is very different from patient one.’ (transcript no. 12)
	^j^ ‘After the second hospitalisation and the schizophrenia diagnosis, I didn’t resign myself to the idea of not having a future any more [red: due to the schizophrenia]. Which was kind of communicated without saying by the HCP, so to say. At least I interpreted it that way, and I didn’t subscribe to that idea.’ (transcript no. 14)
	^k^ ‘There they actually listened to me. And they didn’t work according to the diagnosis, didn’t do everything because of that label.’ (transcript no. 23)
	^l^ ‘I wasn’t even depressed. Depressed is when you’re sad or down, don’t see the point in living. In my opinion, there was an issue within the family that made me stressed out. But the diagnosis they said, it stuck to me during my whole trajectory. I didn’t agree with it, but I couldn’t get rid of it. (…) It felt bad, because I thought; now I’ve suddenly become a psychiatric patient that needs to be stabilised, and no one is focusing on my actual problem. My friends too, I felt stigmatised by them. They couldn’t treat me normally, I felt like a very sick patient when I was with them.’ (transcript no. 5)
	^m^ ‘It was [silence] it was news, it was difficult [silence] to eh, to hear. And at the same time it explained a lot about, well who I am and my position in life. It gave words to a lot of things I already realised, but at the same time, it was very much problematised. It gave me the feeling that I, my whole personality was nothing more than a pile of coping mechanisms.’ (transcript no. 3)

HCP, healthcare professional.

A first relevant dimension in describing diagnosis attitudes and acceptance in this sample was found to be the importance or relevance of the diagnosis (often mentioned as a DSM classification) in someone’s narrative. We saw differences in how often a diagnosis is mentioned in a story, and what meaning is explicitly or implicitly attributed to the diagnosis (e.g. determined as being something significant or insignificant). We read narratives (see Table 3) in which the diagnosis played a significant, key role^a^ in the story, as well as narratives in which the diagnosis was mentioned only briefly^b^ or not at all.^c^

The second dimension that emerged concerned the ‘level’ at which the attitude towards a diagnosis manifested itself. In some stories, this attitude was aimed at the person’s own diagnosis^d^ while in others interviewees spoke about the ‘system’^e^ of diagnosing, or the institution that did the diagnosing. Sometimes, someone’s opinion on both things was consistent;^f^ others disagreed with the system while agreeing with ‘their own diagnosis’.^g^

Their own diagnosis comprised many aspects. The third dimension was on which part of the diagnosis someone’s attitude was based. From the data, we could distinguish between an attitude towards internally originating experiences^h^ such as symptoms and, on the other hand, an attitude towards externally originating experiences such as specific protocols following the diagnosis,^i^ a given prognosis,^j^ experienced stigma, expectations around medication use, access to treatment, access to peer support, practical consequences of a diagnosis, not being listened to^k^ or becoming a ‘patient’ with all its connotations. For some persons, both internal and external factors^l,m^ seemed to be relevant for their opinion.

#### The act of accepting

Many interviewees discussed their attitude towards their ‘diagnosis’ (rather than their DSM-5 classification), which included the aforementioned dimensions. Interviewees often did not explicitly talk about ‘(not) accepting’, but rather used phrases such as ‘coming to terms with’, ‘dealing with’, ‘having closure’, ‘found a place for it’ or ‘having to live with it’. Others actively vocalised their opinion about it rather than undergoing the diagnosis. While some attitudes were expressed in a way that emphasised emotion, others outlined their arguments in a systematic way. Often interviewees would report that their attitudes had changed over time while in other cases the same sentiment could be sensed throughout the whole story. Sometimes people expressed having positive feelings or a sense of pride and empowerment in relation to their situation. For example, one person stated: ‘Well, I am an SMI (severe mental illness) if you want to call it that. I think it’s a badge of honour; an SMI-patient’ (transcript no. 2). Another interviewee stated that ‘If you’re afraid to be a psychiatric patient, you’ll become a psychiatric patient even more, so to say, because you’re [sigh] avoiding, or afraid, whatever. If I say: call me a psychiatric patient, then I can be myself. And then I might not even be within that definition of psychiatric patient any more’ (transcript no. 14).

A noteworthy observation was the use of the term ‘story’ by interviewees: not just in the context of the story they were telling at that moment, but in reference to their broader personal narrative. One interviewee described the importance of being able to speak freely before anyone brings up diagnoses, explaining: ‘My story could be central, without attaching a specific diagnosis or label to it. And that was very important to me’ (transcript no. 20). An aforementioned interviewee found a way of speaking about his mental problems that does justice to himself and his story:

‘How do you view yourself? What do you tell yourself? I often speak of illness, disorder, and vulnerability. My diagnosis is bipolar disorder, but the definition of disorder is that your functioning is impaired and that’s not how I see it. That’s not how I experience it. I’ve been very ill, in the period around that the disorder was acting up. And now I have a vulnerability that sometimes acts up. I think it’s very important in personal recovery, the way you talk about being sick, about illness. You wouldn’t say “I’m a malignant tumour”, but it’s easily said that “You are a schizophrenic, you are a manic-depressive”. The stories people are telling themselves, they can be a barrier towards embracing yourself’ (transcript no. 14).

Like this interviewee, many people spoke about the importance of their story in their recovery process. Another said: ‘A lot of small pieces fell into place, right, now the story is … I feel like I don’t have to dig deeper again’ (transcript no. 15). A previously cited interviewee related that:

*‘*’I read somewhere that a diagnosis should be a sort of story that you create together with a healthcare provider. They look at it from their professional view, and you look at it with your own self-understanding. And I really agree that it should be like that. Most people have some sort of knowledge about themselves, know what they are struggling with. The labelling with a diagnosis, I think it’s so bad’ (transcript no. 5).

## Discussion

### Reflection on principal findings

The objective of this study was to examine key patterns and themes surrounding the acceptance of psychiatric diagnoses, determining to what extent traditional definitions stemming from chronic illness and chronic pain research align with the actual experiences and perspectives of patients. The variability in narrative structure and content was embraced as a source of richness rather than viewed as a limitation, in line with the phenomenological and interpretative frameworks underpinning this study. Our first analysis used existing theoretical concepts related to acceptance and, while applicable to some interviews, we found that these concepts insufficiently captured the meaning in illness narratives. Whereas elements of (non-)acceptance concepts by Williams and Lynn were recognisable, other concepts were deemed not applicable because the texts required substantial interpretation in order for the concepts to fit. In our subsequent analysis, we did not adopt a specific interpretation of diagnosis acceptance nor make general assumptions about it, thereby diverging from previous research in this field, such as studies by Kabzínska-Milewska et al, Pereira et al and de Oliveira et al.^4,5,30^ We then studied the texts for overarching themes concerning diagnosis acceptance and related subjects. Attitudes towards a diagnosis span across and originate in multiple dimensions, and people mostly used terms other than ‘acceptance’ to describe them. Within the main themes related to acceptance of a schizophrenia diagnosis identified by Howe et al, a distinction similar to the one we make – between internal and external dimensions of diagnosis attitudes – can be discerned.^6^ Furthermore, interviewees expressed the importance of being allowed to have and express their own story during a consultation, without it being replaced or overruled by a diagnosis. Additionally, people described changing attitudes throughout the stories, suggesting they might continue to change in the future. This aligns with the notion that acceptance is not always linear but rather continually shifting and fluctuating, focusing on different aspects over time.^31–35^ Many of our findings reflect the complexity highlighted in the phenomenological qualitative study by Pallesen et al, which examines the positive and negative aspects of a bipolar disorder diagnosis.^36^ However, unlike their more binary approach (e.g. fitting versus not fitting, stigmatising versus legitimising), our results are organised according to the levels or dimensions on which these experiences occur.^36^ The following sections will consider and interpret our findings, placing them in a broader context to evaluate their significance and implications.

#### Classification versus diagnosis

Within most narratives, people do not speak distinctly of their DSM-5 classification and/or other elements of their ‘diagnosis’ (‘illness’, ‘disorder’, ‘vulnerability’ etc.), but rather seem to use diagnosis to indicate various aspects of their experience. Psychiatric diagnosis is a description of a clinical syndrome, inherently heterogeneou and with varied aetiology, pathology, clinical features, treatment response, course and outcome. Although incorrect, the conflation of the terms classification and diagnosis is understandable: the concept of a psychiatric classification aiding in diagnosis rather than being a diagnosis or an organic disease can feel complicated or counterintuitive. This might in part explain why so many people automatically and consistently view classifications as a disease causing symptoms, rather than the naming of a cluster of symptoms.^37–40^ It seems that psychiatry as a field is consistently unable to accurately convey some of its most important constructs, and this holds true even in direct patient–clinician relationships.

Nevertheless, our data reveal a distinction between diagnoses and classifications. Within the different dimensions concerning the diagnosis (i.e. more versus less significant, concerning individual diagnosis versus the diagnostic system and internally versus externally originating experiences), only the ‘internally originating experiences’, or clinical signs and symptoms, truly correspond to a DSM-5 classification. Indeed, the classification system is meant to describe a cluster of characteristics – clinical signs and symptoms – and it could be stated that all the other dimensions of attitudes are targeted at the larger, in part culturally constructed, concept of diagnosis. Our findings suggest that the majority of opinions centre around the subjective aspects of this larger concept of diagnosis, that may be summarised as individuals’ perceptions of specific diagnoses. As Jutel wrote, ‘No disease exists in isolation from the way in which it is conferred to those who have the disease’.^41^ Similarly, it could be said that no disease exists in isolation from the way and with which language it is discussed in various settings. It is well known that self-stigma consists of internalised societal convictions (stigma), highlighting the influence of external factors on personal opinions.^42^ Perceptions of diagnoses are shaped by an individual’s upbringing, cultural background, social circles and interactions with HCPs. They are socially constructed, dynamic and can profoundly shape how patients engage with diagnostic labels. Given the myriad influences, unravelling how, where or when exactly these perceptions are formed is challenging. Importantly, some (parts of) interviewees’ perspectives are positive, confirming statements by other authors on the fact that psychiatric diagnoses are not necessarily detrimental to a good life.^43–45^

These findings indicate that only a small portion of opinions and attitudes pertain to DSM classifications, while the majority focus on diagnosis perceptions that are largely outside of psychiatry’s control. This observation aligns with criticisms that raise questions about whether DSM-5 classifications should, or even can, serve as the sole foundation for understanding mental health issues.^46–51^

#### Diagnosis as truth versus alternative views

Existing research often positions a diagnosis as something ‘true’ that must be accepted, with ‘diagnosis acceptance’ typically seen as necessary before treatment, based on the belief that ‘acceptance’ leads to better outcomes than ‘non-acceptance’.^52–55^ In clinical practice, disagreement about a diagnosis may be viewed as lacking illness insight, often described as a ‘symptom’ of various mental disorders, despite illness insight being a highly subjective concept and ignoring the potential negative consequences of labelling problems as ‘mentally ill’.^56^ Moreover, research does not support that diagnosis denial and a lack of illness insight are, per se, connected, although they may co-occur.^57–59^

Our data showed that there are people who do not agree with the medical diagnosis they received and do not use the diagnosis themselves. Nevertheless, they have a good relationship with their HCP. Although many people had strong negative opinions about their diagnosis or the diagnostic system, only one case led to a breakdown in the patient–provider relationship. Even among those who disagreed with the DSM-5 classification of their condition, all acknowledged, along with their HCP, that they had mental problems and needed help or guidance. In the literature, becoming ill or receiving a diagnosis is described as a ‘real situation that must be addressed’,^23,60^ which is what interviewees in our sample overwhelmingly treat it as. Some interviewees preferred and used terms such as ‘highly sensitive person’, ‘complex post-traumatic stress disorder (PTSD)’ and ‘religious trauma syndrome’ over DSM-5 terminology, but treated them as diagnostic terms. Literature supports the findings that alternative explanations for symptoms do not necessarily lead to treatment refusal and, in many cases, people are willing to cooperate.^61^ We believe our findings challenge the dominant assumption that diagnosis acceptance is a prerequisite for successful treatment. Rather, they highlight the central importance of a strong therapeutic relationship, which is in line with previous findings by Pallessen et al.^36^

#### Self-narratives versus diagnosis narratives

Interviewees often emphasised that their personal story, situation and problems needed to be heard before being countered with a diagnosis. The way in which interviewees described this story resembles the philosophical and psychological concept of a self-narrative, that may be described as an ongoing (auto)biography to which the person in question, as well as others, contribute.^31,59,62–64^ Concordantly, the aforementioned perceptions or unique interpretation of a diagnosis – influenced by cultural and societal factors yet individually fine-tuned – are similar to an illness representation or illness model, and could also be considered a ‘diagnosis narrative’.^65–66^ Certain diagnosis narratives can be held at an individual, cultural or societal level. The feelings of discomfort that interviewees reported as resulting from the diagnosis being posed as a challenge to their own self-narrative could therefore be imagined as a clash of narratives^67^ or narrative contestation.^68^ Although the concept of a clash between a diagnosis narrative and the self-narrative has previously been conceptualised,^59,69^ our study is, to our knowledge, the first to frame disagreement about diagnosis as a clash of narratives between patients and professionals, rather than as individual ‘non-compliance’. One interviewee – cited previously – mentioned that she believed a diagnosis should be a collaborative effort between herself and her HCP. Research by Robinson et al supports this, showing that, in the case of dementia, a joint narrative construction with their HCP helped the person and their partner make sense of the diagnosis.^70^ Similarly, Hackmann et al argued that effective collaboration in the diagnostic process could diminish the potential negative consequences of a diagnosis.^71^ Such co-constructed narratives that integrate the diagnosis narrative into the person’s self-narrative might offer an alternative to the clashing narratives that occur when a diagnosis must be ‘accepted’. Narrative negotiation,^72,73^ the process of comparing or constructing elements within a single or shared narrative in order to allow it to develop to its full potential, could be beneficial in this context. Narrative negotiation in the clinical setting involves first the acknowledgement that the HCP and patient are engaging in an act of narrative co-construction. Both parties have something to contribute to the narrative, and both have something at stake. Narrative negotiation thus can be part of the process of co-construction.

### Limitations

Although our approach deliberately diverges from much of the existing literature that conceptualises ‘acceptance’ as a relatively uniform or binary phenomenon, we do believe that people within different diagnostic categories may relate differently to their diagnosis. In our study, however, the sample size for individual diagnostic categories was too small to draw conclusions about specific diagnoses. Another notable limitation is the issue of representation and generalisability due to the selection of participants in the Psychiatry Story Bank project. As noted by van Sambeek et al, the sample consists of only Dutch people and is furthermore moderately homogeneous, potentially skewing the perspectives shared.^17^ The Psychiatry Story Bank project’s ethos, aligned with the recovery movement, might attract participants less inclined towards traditional DSM-based perspectives, which introduces further bias. Additionally, participants are often individuals who have reflected on their past and are more prepared to share their stories, which might not accurately reflect the experiences of patients currently undergoing a turbulent diagnostic process. Finally, the research design’s reliance on self-directed storytelling prevents epistemic injustice but creates some challenges: it limits the ability to pose specific questions, leading to interpretative challenges. If an interviewee does not address certain topics, it may be unclear whether this meant that this subject was not relevant to them, or whether the omission was a deliberate choice, or refusal to use a term or discuss a certain issue. Fully exploring these nuances requires the ability to ask follow-up questions, which was not possible in this research design, potentially leading to different interpretations than intended by interviewees. Furthermore, this means that narratives were not focused specifically on diagnosis acceptance. Nonetheless, 70% of the narratives provided substantial material on acceptance and diagnostic experience, with an additional five people discussing acceptance of diagnosis-related subjects. This suggests that, despite voluntary participation, a significant part of the data-set directly addressed the research questions. It can also be argued that individuals who use their diagnosis to describe themselves refer to it as something applied to them, or treat it as an explanatory model – without explicitly reflecting on their opinion of it – have implicitly or automatically accepted the diagnosis. This applied to eight of the nine participants who did not directly discuss acceptance. Lastly, since our aim was not to measure acceptance across groups, we considered all participants’ narratives to be relevant to our research.

### Implications for research and practice

Our findings underscore the complexity and subjectivity inherent in the (non-)acceptance of psychiatric diagnoses, suggesting the need for even greater sensitivity to the fragmented, dynamic and context-dependent nature of acceptance processes, particularly in psychiatry. Reflecting on the limited practical application of existing theoretical concepts of (non-)acceptance, along with our findings that revealed numerous contextual factors relevant to psychiatric diagnosis attitudes, the relevance and validity of approaching diagnosis ‘acceptance’ as an isolated concept must be carefully re-evaluated. Furthermore, it became apparent that disagreement about diagnostic classifications did not preclude effective collaboration and a strong therapeutic relationship. These points suggest that mutual understanding, as well as finding a shared language for the matters at hand, appear to be more crucial than diagnosis ‘acceptance’.^74^

First, to avoid misunderstandings, clinicians should place more emphasis during the diagnostic process on explicitly communicating the provisional and descriptive nature of psychiatric classifications, rather than presenting them as definitive explanations of underlying causes. Psychoeducational materials and conversations could include clarifications about the distinction between classification and diagnosis as it exists in somatic medicine (implied causal interpretation), and address common misconceptions about biological essentialism or deterministic interpretations of psychiatric labels. To further foster mutual understanding during a diagnostic process, it is essential to understand individuals’ perceptions of their diagnoses and mental problems, as well as the diagnosis narrative they hold. While spontaneously told stories – such as interview transcripts – provide glimpses of these perceptions, they do not clarify exactly what a diagnosis narrative looks like or how it is formed, indicating that this needs to be a topic of discussion in consultation rooms. The understanding of a diagnosis narrative that someone may hold, along with comprehension of their perception of themselves, or self-narrative, is essential for both the individual and the HCP in comprehending the emotional charge and meaning of a (potential) diagnosis. Shared understanding concerning the meaning and implications of a classification or diagnosis, co-constructing a joint narrative and preventing competition between narratives are crucial steps.

To aid in these developments, future research should thus study questions surrounding psychiatric diagnoses and attitudes towards them using narrative approaches, taking context into account. Ongoing research by our team is exploring this context, particularly the way in which someone’s cultural and social context determines explanatory models and perceptions about illness and its treatment. It also explores the particular ways in which individuals in different diagnostic categories relate to their diagnoses. Specifically, the idea of a conflict between diagnosis narratives and self-narratives needs more thorough exploration. Moving forward, efforts to enrich our understanding of acceptance should prioritise exploring diverse narratives and perceptions, as well as study and promote strategies for creating co-constructed narratives. According to our findings, consultations may benefit from being organised not with the acceptance of a diagnosis as an end goal, but rather as an open space where multiple perspectives and narratives can exist and be explored. This approach contributes to greater reciprocality in patient-centred mental health care and research, and perhaps even to health improvement.^75^

In conclusion, our examination of (non-)acceptance in the context of patients’ experiences of psychiatric diagnosis reveals several key insights. Concepts from the literature on diagnosis acceptance are often insufficient or inappropriate at capturing lived experience, as reflected in the analysis of personal narratives. The understanding and interpretation of both the psychiatric diagnosis itself and the concept of acceptance are inherently individualised, highlighting the inadequacy of a simple ‘model’ or theory of acceptance to encompass the complexities of psychiatric vulnerability. Attitudes towards a diagnosis encompass multiple dimensions and are influenced by individuals’ perceptions, although we do not always understand how or where these perceptions are formed. Investigating these aspects to form a deeper understanding is essential in both research and practical individual clinical contexts. The necessity of diagnosis (or DSM classification) acceptance in fostering effective patient–clinician collaboration is uncertain in both existing literature and our empirical data. More important is the patient’s request for a co-constructed narrative, rather than having one imposed by the diagnosis. Rather than emphasising agreement with clinicians, a more fruitful approach might involve exploring patients’ unique experiences through their self-narratives and understanding the content and origin of the diagnosis narratives they hold. We urge researchers and clinicians to explore questions around diagnosis and diagnosis integration using a narrative approach.

## Supporting information

de Rooy et al. supplementary material de Rooy et al. supplementary material

## Data Availability

The data that support the findings of this study are available on request from the corresponding author, M.J.d.R. The data are not publicly available due to their containing information that could compromise the privacy of research participants.

## Supplementary material

The supplementary material is available online at https://doi.org/10.1192/bjo.2025.10810
