# Supplementary material for: The meaning of ‘acceptance’ of a psychiatric diagnosis: qualitative study of illness narratives with review of the literature
Source: BJPsych Open. 2025 Sep 4;11(5):e194. doi: 10.1192/bjo.2025.10810 (PMC12451724; doi:10.1192/bjo.2025.10810)
Supplement: de Rooy et al. supplementary material [file S2056472425108107sup001.docx]

Supplementary Materials to “The meaning of ‘acceptance’ of a psychiatric diagnosis - A qualitative study of personal narratives” by Magali J. de Rooy, Megan M. Milota, Stefan M. van Geelen, Léon C. de Bruin, Floortje E. Scheepers.

**Table of contents**

Table 1: Narrative summary template used in analysis: 2-3

Table 2: Interviewee characteristics 4

Figure 1: Visualization of different dimensions of diagnosis acceptance 5

Table 1: Transcript report used in analysis (translated from Dutch)

| **General information** | |
| --- | --- |
| Researcher’s name |  |
| Date of Story Bank interview |  |
| Gender of respondent (if explicit) |  |
| Age of respondent (if explicit) |  |
| Diagnosis/classification of respondent |  |
| Time between receiving diagnosis/classification and interview, approximately: <1 year, 1-5 years, >5 years |  |
| Have any diagnoses/classifications changed or been withdrawn? Describe. |  |
| **Exploratory questions** | |
| Does this respondent speak about 'acceptance' (or synonyms for it), and acceptance of what, and what experiences? Provide representative quotes that reflect this. |  |
| What is the respondent’s attitude towards the diagnosis/classifications? Are these their own diagnoses, or diagnoses (DSM-5 classifications) in general? Provide representative quotes that reflect this. |  |
| To what extent has there been a change in attitude over time (e.g., immediately after diagnosis vs. at the time of the interview)? Is this attitude constant, or does it fluctuate? Provide representative quotes that reflect this. |  |
| What reasons or examples does the respondent give for their attitude towards the diagnosis? Consider both positive and negative aspects of receiving this diagnosis. Provide representative quotes that reflect this. |  |
| How did it feel for this respondent to receive the diagnosis? How does it feel to have the diagnosis? Provide representative quotes that reflect this. |  |
| To what extent is this respondent (dis)satisfied with the diagnostic process? Why, about what, and based on what? Provide representative quotes that reflect this. |  |
| What terminology does this respondent use regarding the diagnosis? Is it consistent, or are there noticeable variations? Provide representative quotes that reflect this. |  |
| Describe the use of notable examples/comparisons/metaphors/symbolic language: how is reference made to a diagnosis, 'being mentally ill', others with the same diagnosis, etc.? Provide representative quotes that reflect this. |  |
| **Theoretical concepts** | |
| To what extent are Williams' concepts of acceptance recognizable? Nonattachment / Nonavoidance / Nonjudgment / Willingness / Tolerance |  |
| To what extent are Williams' concepts of nonacceptance recognizable? Denial / Avoidance / Escape / Prejudice / Noncompliance |  |
| To what extent are concepts of active and resigning acceptance recognizable? Nonacceptance / Active Acceptance / Resigning Acceptance |  |
| To what extent is receiving a diagnosis and the subsequent period discussed as a grieving process? Provide representative quotes that reflect this. |  |
| To what extent is there discussion of a process or change in dealing with the diagnosis? Are concepts like 'resilience' and 'adaptation' recognizable? |  |
| **Summarizing questions** | |
| Is there acceptance or nonacceptance of diagnosis/classifications in the broadest sense? Provide a brief summary. |  |
| What role does the diagnosis play for this respondent? |  |
| To what extent is there 'accordance,' or agreement reached between the respondent and others (e.g., healthcare provider, relatives) about the diagnosis, and in what manner? Provide a brief summary. |  |
| **Researcher’s remarks** | |
| Impressions and observations during the reading |  |
| Emerging questions during the reading that could potentially contribute to broad interpretation/analysis:   - Was there a 'trigger' for a change in acceptance or attitude? - Does the respondent discuss disclosure of the diagnosis to others: to whom, in what manner, and in what setting or context? - What was the lead-up to the diagnosis? Self-initiated, advised by others, ended up in psychiatry due to the severity of symptoms, etc. |  |
| Other (e.g., points that do not fit above but are important to the essence of the story) |  |

| **Gender** | **Interviewees % (n)** |
| --- | --- |
| Male | 33·33% (10) |
| Female | 56·66% (17) |
| Not indicated or unclear | 10% (3) |
| **Age (reported or reasonably certain estimate)** | **Interviewees % (n)** |
| Young adult (18-30) | 23·33% (7) |
| Middle age (30-60) | 60% (18) |
| Older age (60+) | 10% (3) |
| Unclear | 6·66% (2) |
| **Diagnosis** | **Number of individual diagnoses** |
| Addiction | 2 |
| ADHD | 2 |
| Anxiety disorder | 1 |
| Autism Spectrum Disorder | 4 |
| Bipolar disorder | 7 |
| Depression | 4 |
| Dyslexia | 2 |
| Eating disorder | 2 |
| Obsessive Compulsive Disorder | 2 |
| Personality disorder | 5 |
| Post Traumatic Stress Disorder | 3 |
| Psychosomatic disorder | 1 |
| Schizoaffective disorder | 1 |
| Schizophrenia | 1 |
| Non-DSM term | 15 |
| **Number of diagnoses reported (not including non-DSM terms)** | **Interviewees % (n)** |
| 0 | 10% (3) |
| 1 | 53·33% (16) |
| 2 | 33·33% (10) |
| 3 | 3·33% (1) |
| **Terminology use** | **Number of individuals mentioning** |
| ‘Diagnosis’ | 30 |
| ‘Classification’ | 1 |
| Any DSM-5 classification | 29 |
| ‘Susceptibility’ language (e.g. ‘susceptible to psychosis’) | 2 |
| Other categories (e.g. ‘burnout’, ‘highly sensitive person’ ‘highly gifted’, ‘complex grief,’ ‘complex PTSD’, ‘attachment problem’) | 10 |
| **Mental healthcare status at time of interview** | **Interviewees % (n)** |
| Some form of mental healthcare | 86·66% (26) |
| No mental healthcare | 10% (3) |
| Unclear | 3·33% (1) |
| **Reason for switch/end of mental healthcare initiated by interviewee** | **Interviewees % (n)** |
| Disagreement about diagnosis | 3·33% (1) |
| Other or unknown | 10% (3) |
| No switch or ending mentioned | 83·33% (25) |

Table 2: Interviewee characteristics


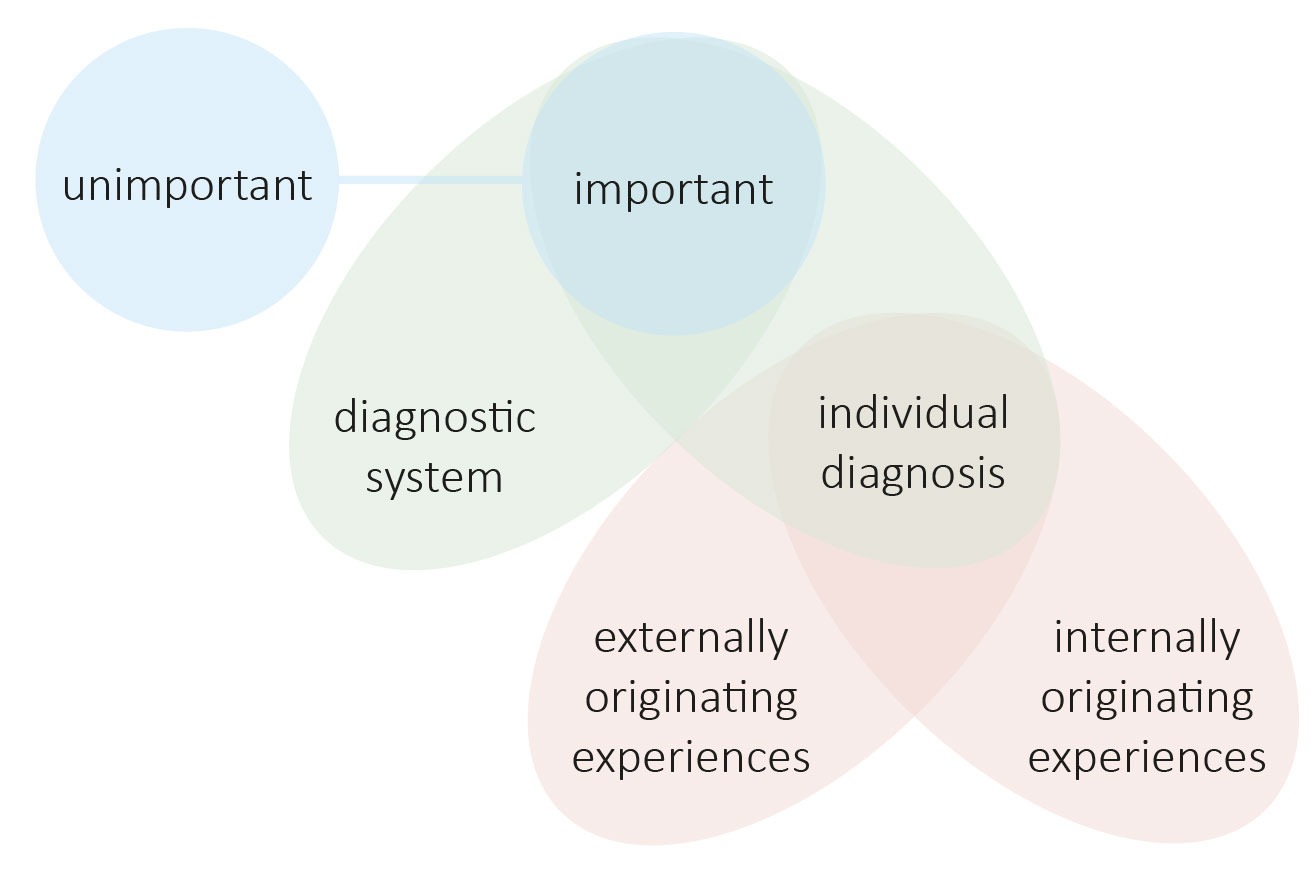


Figure 1: Visualization of different dimensions from which diagnosis attitudes can originate
